# Synovial fluid pH is as specific as synovial leukocyte count but less sensitive for the diagnosis of chronic prosthetic joint infection

**DOI:** 10.1186/s10195-022-00672-5

**Published:** 2022-11-19

**Authors:** Christoph Theil, Thomas Ackmann, Georg Gosheger, Jan Puetzler, Burkhard Moellenbeck, Jan Schwarze, Martin Schulze, Sebastian Klingebiel

**Affiliations:** Department of Orthopedics and Tumour Orthopedics, Muenster University Hospital, Albert-Schweitzer-Campus 1, 48149 Muenster, Germany

**Keywords:** Periprosthetic joint infection, Revision hip arthroplasty, Revision knee arthroplasty, Synovial fluid

## Abstract

**Background:**

Synovial fluid markers (synovial leukocyte count and differential) have been shown to be most accurate in diagnosing prosthetic joint infection (PJI). An inverse correlation for synovial leukocyte count and synovial pH is known assuming that leukocyte metabolism causes synovial fluid acidosis. This study’s purpose is to analyze the use of synovial pH as a potential diagnostic marker for PJI.

**Materials and methods:**

92 patients who presented with painful total joint arthroplasty (TJA) of the hip (THA; *n* = 25) or knee (TKA, *n* = 67) were prospectively investigated. In our cohort 33% (30/92) had PJI and 67% (62/92) were diagnosed non-infected based on the modified Musculoskeletal Infection Society (MSIS) criteria of 2018. Receiver operating curves and the Youden’s index were used to define an ideal cut-off value for synovial pH and the sensitivity and specificity were calculated using cross-tables. Additionally, the sensitivity and specificity were calculated for synovial white blood cell (WBC) count (cut-off > 3000 leukocytes) and percentage of neutrophils (PMN%, cut-off > 80%).

**Results:**

The median synovial pH level was significantly lower in the group with chronic PJI compared to implants with aseptic failure (7.09 vs. 7.27; *p* < 0.001). The calculated optimal cut-off value was 7.11 (AUC 0.771) with a sensitivity of 53% and specificity of 89%. However, the sensitivity and specificity of synovial WBC count were 90% and 88% and for synovial PMN% 73% and 98%, respectively.

**Conclusion:**

Synovial pH may be a useful adjunct parameter in the diagnosis of chronic PJI after hip or knee arthroplasty, but showed low sensitivity in this preliminary cohort. Future studies with larger numbers are needed.

**Level of evidence:**

2a, diagnostic study.

*Trial registration* German Clinical Trials Register (Registration number: DRKS00021038).

## Introduction

Periprosthetic joint infection (PJI) is one of the most feared complications after total joint arthroplasty (TJA) of the hip (THA) or knee (TKA) with an incidence of 1–2% [1–3]. Due to the increasing number of performed joint replacements, the number of revision arthroplasties due to PJI will continue to rise in the coming years [4–6]. Timely and accurately diagnosing of PJI—especially in chronic cases—is still challenging in daily clinical practice and since to date no optimal, universal test is available [6, 7].

Currently, the diagnosis of PJI is usually established using the criteria published by the Musculoskeletal Infection Society (MSIS) and by the International Consensus Meeting (ICM) proceedings [8, 9]. Beside the clinical findings as well as serum and microbiological testing, both definitions include synovial white blood cell (WBC) count and percentage of synovial polymorphonucleocytes (PMN%). Synovial fluid analysis appears to be most reliable at the moment [10, 11] but still has no perfect diagnostic accuracy and the optimal threshold for different joints and organisms are debated [9, 12, 13].

The potential role of synovial fluid pH as a biomarker to differentiate identify joint with inflammatory joint disease and those with normal joints was investigated (*n* = 16) as early as 1966 [14]. Generally, the pH scale is used to specify the acidity or basicity of an aqueous solution [15]. Acidic solutions (solutions with higher concentrations of H^+^ ions) have lower pH values than alkaline solutions. Cummings et al. [14] described a significantly lower synovial pH value in a small series of 16 patients with inflammatory joint disease compared to non-inflammatory joints (7.223 vs. 7.434). Later, Ward et al. demonstrated a close correlation for an increasing synovial WBC count and an lowered synovial pH in 22 patients native joint with septic arthritis [16]. This inverse correlation of WBCs and pH suggests that white blood cell metabolism is responsible for synovial fluid acidosis. Considering that PJI is associated with an increased synovial WBC count [8, 9], this study evaluates synovial pH in patients undergoing revision arthroplasty of the hip or knee as a potential, preoperative synovial biomarker for chronic PJI and compares its accuracy with synovial WBC count and PMN%.

## Materials and methods

The study was registered in the German Clinical Trials Register (Registration number: DRKS00021038) and approval of the institutional review board of the authors’ institution was obtained prior to this investigation (local ethical committee ref. no. 2019-666-f-S, approval granted 26.02.2020). A specific source of funding was not required for this study. It was conducted according to the principles of the World Medical Association Declaration of Helsinki, and written consent was given by all participants.

All patients who presented to our tertiary center for revision arthroplasty with a potential indication for revision either for chronic PJI (symptoms greater six weeks) or for aseptic causes routinely undergo tests of serum, synovial and microbiological parameters prior to surgery in order to rule out PJI. For this study we added a synovial pH-test to the diagnostic preoperative algorithm and analyzed data collected between August 2019 and October 2020. Synovial fluid was collected in safePICO syringes (Radiometer GmbH, Krefeld, Germany) with a built-in mixing ball and dry electrolyte-balanced heparin, helping to minimize the risk for clot formation and measured by potentiometry on a commercial blood gas analyzer (ABL 90 FLEX PLUS, Radiometer GmbH, Krefeld, Germany).

For cell count and differential, synovial fluid was obtained at the time as for pH analysis and stored in an ethylendiamintetraacetic acid screw cap tube (S-Monovette, Sarstedt AG, Numbrecht, Germany). Total cell counts and percent of polymorphonuclear/neutrophile leukocytes were analyzed in synovial fluid with an automatic Sysmex XN-9000 hematology analyzer (Sysmex Corporation, Kobe, Japan) on body fluid mode. Synovial fluid for microbiology culture was stored in screw cap tube without additives (S-Monovette, Sarstedt AG, Numbrecht, Germany) and immediately transported to the microbiology laboratory.

In our practice, all patients undergo analysis of serum C—reactive protein (CRP), serum interleukin-6 (Il-6), serum and synovial WBC count, differentiation of synovial WBCs including PMN% and synovial microbiology culture. We excluded patients with chronic systemic inflammation such as rheumatoid arthritis, those who had surgery within the last 4 weeks, with malignancies, with confirmed inflammatory diseases of other organs such as pneumonia and urinary tract infection. For patients that underwent revision arthroplasty (*n* = 62), either for chronic PJI (*n* = 30) or aseptic failure (*n* = 32), a minimum of five intraoperative tissue samples for microbiology cultures were taken additionally to the synovial fluid, and another tissue sample was taken for histological analysis. All tissue and synovial fluid samples were cultured for a minimum of 14 days on Columbia blood agar, chocolate agar and Schaedler agar (both containing 5% sheep blood; BD Sparks, MD, USA). Additionally, enrichment in brain–heart infusion broth for ten days and thioglycollate medium (BBL™; BD, Sparks, MD, USA) was performed. Furthermore, 34 patients were evaluated preoperatively, but did not undergo surgery so far because they declined surgery at this point or underwent surgery elsewhere or a non-operative management has been recommended. Therefore, 96 patients were eligible for analysis. Participants meeting the study’s inclusion criteria were prospectively evaluated and classified as infected (*n* = 30), not-infected (*n* = 62) or inconclusive (*n* = 4) as defined by the modified MSIS criteria of 2018. Those with an inconclusive score were excluded from the statistical analysis. In total 92 patients (25 THA, 67 TKA, 46 male, median age 69 (IQR 60—76) were included in this study. Thirty patients (19 TKA, 11 THA) were assigned to the PJI group, while the remaining 62 patients were not considered infected (48 TKA, 14 THA).

### Statistical analysis

Data collection and statistical analysis were performed using Excel (Microsoft Corporation, Redmont, Washington, USA) and Statistical Package for the Social Sciences Statistics for Windows version 25 (IBM Corporation, Armonk, NY, USA). All patient records were anonymized prior to analysis. Descriptive statistics and the Shapiro–Wilk test were used to analyses distribution of data. The means and ranges were calculated for parametric data; the medians and 25–75% interquartile ranges (IQRs) were obtained for non-parametric data. The non-parametric analyses were performed using the Mann–Whitney U test. Frequencies were given for categorical variables that were compared in contingency tables using the chi-squared test.

Statistical evaluation was performed with receiver operating characteristics (ROC) analyses with presentation of the area under the curve (AUC) with 95% confidence interval (CI). The Youden’s index was used to determine the optimal cut-off value for synovial pH. Based on the determined cut-off value for synovial pH and the given cut-off values for WBC count (> 3000/µl) and PMN% (> 80%) [8, 9], sensitivities and specificities were calculated for each biomarker from contingency tables. Statistical significance was set at *p* ≤ 0.05, all p-values were two-sided.

## Results

The median synovial pH level was significantly lower in the patients diagnosed with chronic PJI [7.09 (IQR, 6.98–7.26)] compared to the patients with aseptic failure [7.27 (IQR, 7.17–7.32)] (*p* < 0.001).

**Fig. 1:**
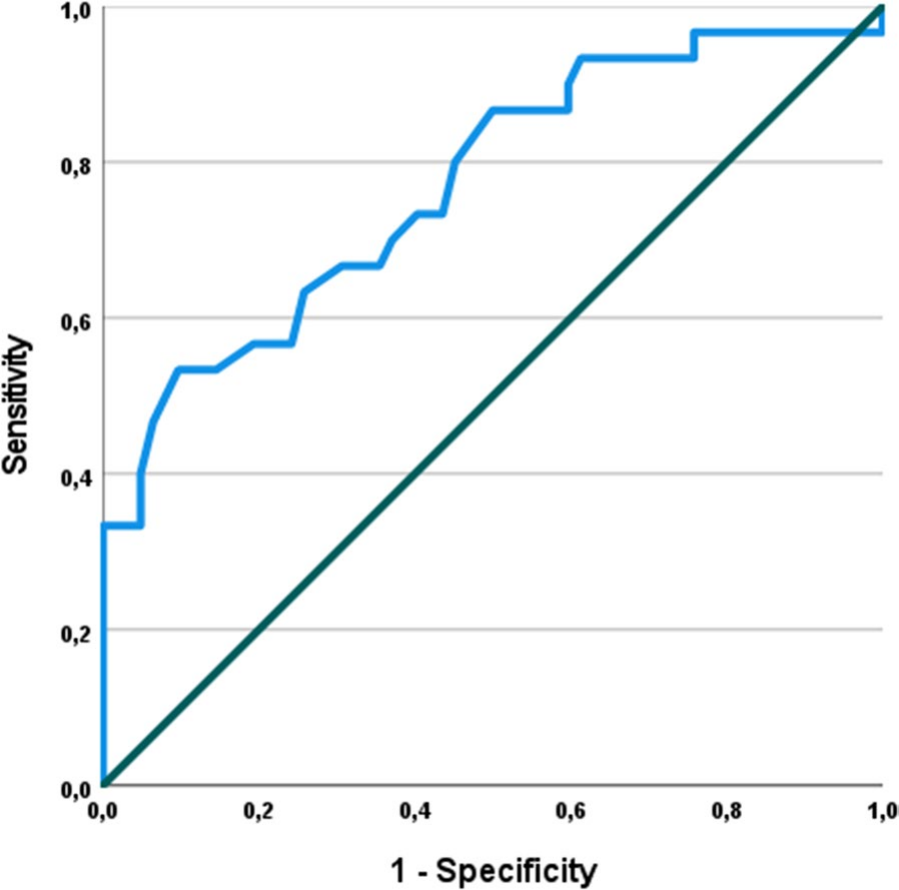
Receiver operating characteristics curve for synovial pH

The ROC curve analysis showed an AUC of 0.771 [95% confidence interval (95% CI), 0.664–0.878] (Fig. 1) for synovial pH. Using Youden’s index, the optimal cut-off value was 7.11 for synovial pH discriminating between chronic PJI and aseptic failure. Fifty-five of 62 not infected samples had a synovial pH value above 7.11 and 16 of 30 infected samples had a synovial pH below 7.11. Applying this threshold, a specificity of 89% and sensitivity of 53% were calculated. The positive predictive value to correctly identify chronic PJI was 69% and the negative predictive value was 79% (Table 1).

**Table 1:** Results for synovial pH value, synovial white blood cell (WBC) count and percentage of synovial neutrophils (PMN%) in diagnosing prosthetic joint infection (PJI)

Markers	Used cut-off values	Sensitivity (%)	Specificity (%)	PPV	NPV
pH	< 7.11	53	89	0.6956	0.7971
WBC count	> 3000/µl	90	88	0.7941	0.9464
PMN%	> 80%	73	98	0.8529	0.8787

In comparison, the median level of synovial WBC count (14,905 cells/μL (IQR, 6692–28,898) vs. 660 cells/μL (IQR, 310–1422); *p* < 0.001) and synovial PMN% [90.6% (IQR, 76.6–94.6%) vs. 26.9% (IQR, 19.5–45.3%), *p* < 0.001] were significantly higher in patients with chronic PJI compared to patients with aseptic failure.

The highest AUC was shown for synovial PMN% with 0.962 (95% CI 0.911–1.000), followed by synovial WBC count with 0.936 (95% CI 0.880–0.993). Using the given cut-off values, the sensitivity and specificity of synovial WBC count were 90% and 88% and 73% and 98% for synovial percentage of PMN, respectively.

In 25 of 30 cases (83.3%) with chronic PJI a causative bacterium was identified (Table 2), while five (16.7%) patients had culture-negative infection and four (13.3%) presented with a polymicrobial infection. Among the patients with culture-negative infection, one patient had a fistula and the other cases each had an elevated CRP, elevated synovial WBC count and elevated synovial PMN%. There were no unexpected positive cultures in the group that was considered not infected preoperatively.

**Table 2:** List of the identified bacteria for the 25 patients the PJI group

Culture organism	Frequency (%)
*Staphylococcus lugdunensis*	3.8
*Enterobacter cloacae complex*	5.8
*Corynebacterium*	3.8
*Staphylococcus epidermidis*	17.2
*Escherichia coli*	3.8
*Streptococcus agalactiae*	3.8
*Enterococcus faecalis*	7.7
*Staphylococcus caprae*	3.8
*Staphylococcus aureus*	3.8
*Staphylococcus capitis*	7.7
*Streptococcus dysgalactiae*	3.8

## Discussion

PJI continues to affect a remarkable number of patients who undergo TJA but the accurate diagnosis is still challenging in daily clinical practice and the search for potential serological and synovial biomarkers is ongoing [17–19]. This study evaluates synovial pH as a potential novel marker for chronic PJI. Using the calculated cut-off value of 7.11, synovial pH showed some potential to the diagnostic pathway of chronic PJI with a high specificity (89%), however it had a poor sensitivity (53%). Despite the low sensitivity, we identified a significant difference for the pH value of the chronic septic and aseptic cases after TJA of the knee or hip.

A high synovial WBC and increased production of protons by neutrophils during the activation of the respiratory burst is responsible for decreasing synovial pH [8, 20]. Furthermore, the presence of bacteria leads to high synovial lactate concentrations and this again correlates with a drop of synovial pH [21]. For the present cohort culture negative infection was rare and four patients had polymicrobial infection and in general 11 different organisms were detected which poses the question if and to what extent [21], different bacteria result in varying synovial pH value as it has been reported for native joint infections [21]. Furthermore, previous studies have discussed and proposed different thresholds for leukocyte count and differential depending on the type of infection (low-grade or high-grade) or even individual pathogens. This may also be the case for synovial pH and should be answered in further investigations with larger numbers.

The findings of this study must be interpreted considering several limitations: First, we excluded four patients with inconclusive results based on the modified MSIS 2018 criteria as we felt that in order to evaluate a novel marker, it would be prudent to only include patients where the diagnosis is relatively certain based on validated criteria. However, future studies must elucidate whether synovial pH might be helpful in difficult to diagnose cases. Secondly, while a correlation between synovial leukocyte count and pH appears logical, the sensitivity varies greatly in this study. While the reason is unclear, it is possible that with the numbers available, the calculated cut-off for pH is not optimal and future studies are needed to refute or repeat this result. Furthermore, other leukocyte derived markers such as alpha defensine and leukocyte esterase should be included as well. Thirdly, 34 patients in the study cohort did not undergo prosthesis exchange, but were classified as infected or non infected based on preoperative diagnostics. Among those, 12 patients had unexplained pain and might therefore have infection that was missed by the diagnostic algorithms. Fourthly, as this is a study focusing on preoperative diagnosis, it was not the aim of the study to define a minimum follow-up period and it is possible that among those patients who did not undergo surgery at the authors’ hospital at the time when the study was performed, some were diagnosed with infection later on. Therefore, it would be interesting to see how the diagnostic biomarkers develop over time in these patients.

In addition to the other possible influencing factors such as gender, age, affected joint [22] or comorbidities, the size of the implanted endoprosthesis can be of relevance and needs further investigations. Milošev et al. observed a small but statistically significant difference in the pH of synovial fluid between natural joints with degenerative diseases and joints with metal implants [22]. It has been hypothesized that the release for metal ions from joint implants may lead to a decrease in synovial pH. Therefore it is possible that the pH may be influenced by implant size or material with megaprosthesis potentially leading to a lower synovial pH and potentially lower diagnostic threshold. Furthermore, a long-lasting TJA may have released more metal ions over time due to mechanical wear which might also lower the synovial pH. However, the usefulness of synovial cell count and differential has been generally questioned in this context when a form of metallosis or adverse local tissue reactions is present. Nonetheless, to our knowledge the effect of implant size on synovial leukocyte count and differentiation has not been explored to the author’s knowledge although a difference is possible. Therefore, future studies should also investigate synovial pH if increased metal ion release is present (metal-on-metal bearing, metallosis, megaprostheses, long-lasting implants) in conjunction with synovial leukocytes.

Furthermore, most recently Abdelaziz et al. [23] investigated differences in synovial cell count for different aseptic reasons for revision arthroplasty in 702 cases of revision THA. They found that for some indication such as polyethylen wear or osteolysis, common diagnostic threshholds in cell count for PJI can’t be applied and that im some instances cell count might be greater than 7000 leuocytes per μl. Therefore, in such cases other synovial markers might be needed. It is unclear whether synovial pH might be advantageous in these cases and future studies should investigate synovial pH in this context.

The combination of microbiological long-term incubation, leukocyte count and leukocyte differentiation is the current gold standard analysis of synovial fluid [12]. Especially WBC count and PMN% provide high sensitivities and specificities as demonstrated in numerous studies [8, 9, 12]. While this is the first study to investigate synovial pH for chronic PJI, synovial WBC count and PMN% have already been examined in numerous previously studies but there is still disagreement among orthopedic surgeons about the optimal threshold [24]. For our investigation we used the thresholds given by MSIS 2018 [8] to determine the sensitivity and specificity. For our cohort the specificity of WBC count (88%) and PMN% (98%) was as high as for pH (89%), whereas the sensitivity was best for WBC count (90%), but lower for PMN% (73%) and pH (53%). However, other institutions [12] or authors [25] recommend lower cut-off values to detect low-grade infections due to the fact, that the change of synovial fluid parameters depends on the virulence of bacteria [12] and may be as low as 1500 leukocytes and 65% PMN which must also be considered when interpreting synovial pH testing and the sensitivity reported here.

## Conclusion

In conclusion, synovial pH could be a helpful adjunct parameter in the diagnosis of chronic PJI, given its reasonably high specificity, particular if cell count was not feasible. However, due to the low sensitivity, this parameter should be combined with other serum and synovial biomarkers. This combination of parameters should be investigated in larger trials and could be particularly helpful in inconclusive cases although further study is certainly needed.

## Data Availability

Raw data is available upon reasonable request.

## Abbreviations

AUC: Area under the curve
ROC: Receiver operating characteristics
IQR: Interquartile range 25–75%
PJI: Periprosthetic joint infection
PMN: Polymorph neutrophiles
THA: Total hip arthroplasty
TJA: Total joint arthroplasty
TKA: Total knee arthroplasty
ICM: International consensus meeting
MSIS: Musculoskeletal infection society
WBC: White blood cell
